# Development of a Semi-Automated, Bulk Seeding Device for Large Animal Model Implantation of Tissue Engineered Vascular Grafts

**DOI:** 10.3389/fbioe.2020.597847

**Published:** 2020-10-23

**Authors:** Eoghan M. Cunnane, Katherine L. Lorentz, Lorenzo Soletti, Aneesh K. Ramaswamy, Timothy K. Chung, Darren G. Haskett, Samuel K. Luketich, Edith Tzeng, Antonio D’Amore, William R. Wagner, Justin S. Weinbaum, David A. Vorp

**Affiliations:** ^1^Department of Bioengineering, University of Pittsburgh, Pittsburgh, PA, United States; ^2^Tissue Engineering Research Group, Department of Anatomy, Royal College of Surgeons in Ireland, Dublin, Ireland; ^3^McGowan Institute for Regenerative Medicine, University of Pittsburgh, Pittsburgh, PA, United States; ^4^Department of Surgery, University of Pittsburgh, Pittsburgh, PA, United States; ^5^RiMED Foundation, Palermo, Italy; ^6^Department of Chemical and Petroleum Engineering, University of Pittsburgh, Pittsburgh, PA, United States; ^7^Department of Pathology, University of Pittsburgh, Pittsburgh, PA, United States; ^8^Department of Cardiothoracic Surgery, University of Pittsburgh, Pittsburgh, PA, United States; ^9^Clinical and Translational Sciences Institute, University of Pittsburgh, Pittsburgh, PA, United States

**Keywords:** vascular tissue engineering, sheep model, carotid implantation, mesenchyaml stem cells, bulk seeding

## Abstract

Vascular tissue engineering is a field of regenerative medicine that restores tissue function to defective sections of the vascular network by bypass or replacement with a tubular, engineered graft. The tissue engineered vascular graft (TEVG) is comprised of a biodegradable scaffold, often combined with cells to prevent acute thrombosis and initiate scaffold remodeling. Cells are most effectively incorporated into scaffolds using bulk seeding techniques. While our group has been successful in uniform, rapid, bulk cell seeding of scaffolds for TEVG testing in small animals using our well-validated rotational vacuum technology, this approach was not directly translatable to large scaffolds, such as those required for large animal testing or human implants. The objective of this study was to develop and validate a semi-automated cell seeding device that allows for uniform, rapid, bulk seeding of large scaffolds for the fabrication of TEVGs appropriately sized for testing in large animals and eventual translation to humans. Validation of our device revealed successful seeding of cells throughout the length of our tubular scaffolds with homogenous longitudinal and circumferential cell distribution. To demonstrate the utility of this device, we implanted a cell seeded scaffold as a carotid interposition graft in a sheep model for 10 weeks. Graft remodeling was demonstrated upon explant analysis using histological staining and mechanical characterization. We conclude from this work that our semi-automated, rotational vacuum seeding device can successfully seed porous tubular scaffolds suitable for implantation in large animals and provides a platform that can be readily adapted for eventual human use.

## Introduction

Cardiovascular disease remains the primary cause of global death and encompasses disorders of the heart and blood vessels (Mendis et al., 2011; Benjamin et al., 2017). The vessels most frequently affected by cardiovascular disease are the coronary and peripheral arteries which require revascularization surgeries to treat occlusion and distal ischemia, respectively (Goodney et al., 2009; Alexander and Smith, 2016). Stent deployment is effective in the revascularization of localized obstructions; however diffuse obstructions require bypass surgery. The saphenous vein and internal mammary artery are the gold standard bypass conduits for small diameter vessels (<6 mm diameter) of the coronary and peripheral arteries, respectively. However, saphenous veins are often unavailable or unsuitable and frequently fail due to intimal hyperplasia (Isenberg et al., 2006), while the failure of mammary artery grafts due to occlusion is a persistent issue (Harskamp et al., 2015). Furthermore, synthetic grafts are not a viable treatment option for small diameter vessels due to high rates of acute thrombosis (Klinkert et al., 2004; Desai et al., 2011).

The advancement of tissue engineered vascular grafts (TEVGs) in recent years, and their ability to form functional neo-vessels, presents as a promising clinical option for the treatment of vascular disease (Cunnane et al., 2018). TEVGs often incorporate cells into biodegradable scaffolds through various cell-seeding techniques (Villalona et al., 2010; Weinbaum et al., 2020), and the presence of cells has demonstrated improved TEVG patency rates through reduced thrombosis and stenosis (Nieponice et al., 2010; Soletti et al., 2011; Krawiec et al., 2016, 2017; Haskett et al., 2018).

In order to achieve homogenous incorporation of cells within TEVG scaffolds, bulk seeding techniques have come to be preferred over the simpler drip or static seeding techniques first used to impregnate scaffolds with cells (Soletti et al., 2006). However, the majority of work to date has focused on seeding TEVGs intended for implant in small animal models (Nieponice et al., 2008; Hibino et al., 2011; Udelsman et al., 2011, 2014). The generation of cell-based, larger sized TEVGs (∼4 mm inner diameter, ∼100 mm length) suitable for testing in large animal models or eventual translation to the clinic requires scale-up of both the scaffold and the bulk seeding system. Additionally, to ensure regulatory approval and effective clinical translation, a semi-automated bulk cell seeding device that can create a reproducible TEVG is required. To this end, the purpose of this work was to develop and validate a semi-automated, rapid, bulk seeding device that results in homogenous cell distribution within large scaffolds intended for use as “human-sized” TEVGs. We demonstrate the utility of the device by bulk seeding a scaffold with adipose derived stromal cells and implanting the resulting construct in a sheep model to examine patency and remodeling over a 10-week period.

## Methods

### Design of the Translating-Rotating Seeding Device

**FIGURE 1:**
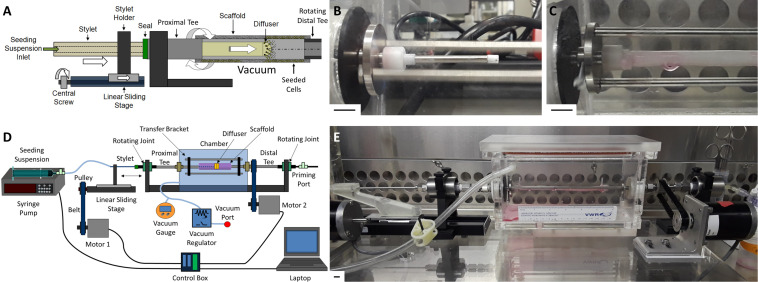
Overview of translating-rotating seeding device. **(A)** Schematic of the linear sliding stage system used to translate the Stylet/Diffuser component within the rotating scaffold lumen during seeding under vacuum. **(B)** Image of the Stylet/Diffuser system emerging from the proximal mounting tee. **(C)** Image of a mounted scaffold with the Diffuser infusing cell suspension within the scaffold lumen. **(D)** Schematic and **(E)** image of the novel cell seeding device developed in this study to bulk seed human-sized tubular scaffolds. Scale bars depict 1 cm.

The design of our novel bulk seeding device for large, “human-sized” scaffolds was first conceived during the doctoral dissertation work of Soletti (2008). It is based on our lab’s previously published and validated custom rotational vacuum seeding device which has been used to produce TEVGs for small animal testing (Nieponice et al., 2010; Soletti et al., 2011; Krawiec et al., 2016, 2017; Haskett et al., 2018). The common concept is to achieve bulk seeding of rotating, porous, tubular scaffolds via local luminal delivery of cells under applied vacuum. The new system presented herein adds a cell-releasing “Diffuser” equipped with eight equi-spaced radial nozzles attached to a “Stylet” arm. The Stylet drives the Diffuser coaxially along the longitudinal axis of the tubular scaffold and transports cell suspension from a syringe, locally to the scaffold lumen while the scaffold rotates under an applied vacuum (Figures 1A,B). The locally delivered cells are drawn into the wall of the scaffold, via the applied vacuum, through the interconnected pore network, to ensure even radial cell distribution (Figure 1C). The linear displacement of the Diffuser ensures even longitudinal cell distribution, while the rotation of the scaffold ensures even circumferential distribution.

Linear translation of the Stylet/Diffuser is achieved using a stepper motor (“Motor 1”) attached to a sliding stage. Rotation of the scaffold is achieved using an additional stepper motor (“Motor 2”) connected to a mounting tee located distally to the scaffold (“Distal Tee”). The Distal Tee is connected to the proximal mounting tee (“Proximal Tee”) via a bracket that transfers the rotational momentum (Figures 1D,E). Two PTFE supports are attached to the ends of the tees to allow for mounting of the scaffold within the vacuum chamber. A syringe pump supplies the cell suspension to the Diffuser, through the Stylet. Both the pump and the motors are controlled using a custom Labview program (National Instruments, TX, United States) that allows for control of the Stylet translation speed, the scaffold rotation speed and the syringe pump infusion rate. Additional detail regarding the device, shear stress on the seeded cells and sterilization of the device is provided in Supplementary Material.

### Scaffold Design

The biodegradable, bi-layered, elastomeric scaffolds used in this study are manufactured from poly(ester urethane)urea (PEUU) as described previously (Nieponice et al., 2010; He et al., 2011, 2010; Krawiec et al., 2016). The porous inner layer of the scaffold was created using thermally induced phase separation in a tubular mold. The inner layer was then coated by electrospinning an additional layer of PEUU for mechanical stability. Scaffolds are tubular, 4.7 mm inner diameter, 5.2 mm outer diameter, and 10 cm in length, to approximate the shape and size of a human coronary or peripheral artery. The structural and morphological properties of the scaffold have been fully characterized previously (Soletti et al., 2010). Briefly, the inner layer of the scaffold has a pore size measuring 123 ± 20 μm (mean ± SD), the outer layer has a pore size measuring 5.1 ± 3.2 μm, while the diameter of the outer layer nanofibers is 743 ± 201 nm. Scanning electron microscope images of the scaffold can be found in Supplementary Material and also in Soletti et al. (2010).

### Bulk Seeding Validation

#### Cell Source and Culture

PEUU scaffolds were bulk seeded with human adipose derived mesenchymal stem cells (hADMSCs) to validate the seeding device. The hADMSCs were obtained commercially (Rooster Bio, Inc., Frederick, MD, RoosterVial-hAD-1M MSC Lot #00097) and cultured in supplemented growth media (RoosterBio, SU0005, GM) until passage 2. The hADMSCs were then frozen in freezing media [90% FBS (Atlanta Biologics) and 10% DMSO (Sigma)] until ready for use. For each seeding study, a stock of 1 million hADMSCs was thawed into a 175 cm^2^ flask (Falcon) and cultured for 3 days in 15 mL of GM. Media was replaced after 16 h to remove residual DMSO. After 3 days of culture, 6 million cells were passed into two 5-layer tower flasks (equivalent to approximately 3,500 cells/cm^2^) and cultured for 72 h in 75 mL of GM. The cells were then passed into 10, 5-layer tower flasks and cultured for a further 72 h in 75 mL of GM per flask to obtain approximately 200 million cells for seeding.

A cell number of 200 million was chosen as it approximates the cell density (cell number per volume of scaffold material) employed in our previous small animal studies. Those studies demonstrated that a cell density in excess of 400 cells per cm^3^ of scaffold material prevents acute thrombosis and initiates positive scaffold remodeling upon implantation (Krawiec et al., 2017; Haskett et al., 2018). We therefore targeted a cell density in excess of 400 cells per cm^3^ of material as our cell seeding density, which requires approximately 200 million cells for a 12 cm scaffold with 4.7 mm inner diameter and 5.2 mm outer diameter. The calculations used to determine cell seeding density are provided in Supplementary Material.

#### Cell Seeding

The performance of the cell seeding device was examined by seeding PEUU scaffolds (10 cm in length) with approximately 200 million hADMSCs suspended in 30 mL of GM and characterizing the longitudinal and circumferential distribution of cells in the seeded scaffold. Linear translation speed was varied (2.5, 5, and 7.5 mm/s) to examine the effect of diffuser displacement rate on cell distribution. The dispensed volume was kept consistent across displacement rates by varying the number of complete cycles through the scaffold lumen (2, 4, and 6 cycles for 2.5, 5, and 7.5 mm/s, respectively). The remaining seeding parameters were kept constant (flow rate = 12.5 mL/min, rotation speed = 60 rpm, and applied vacuum = −127 mmHg). Seeding efficiency was calculated using the following expression (Wendt et al., 2003; Zhao and Ma, 2005):

$$\frac{\begin{array}{c} CellNumberinSuspensionBeforeSeeding\\ -CellNumberinSuspensionAfterSeeding \end{array}}{CellNumberinSuspensionBeforeSeeding}\times 100.$$

**FIGURE 2:**
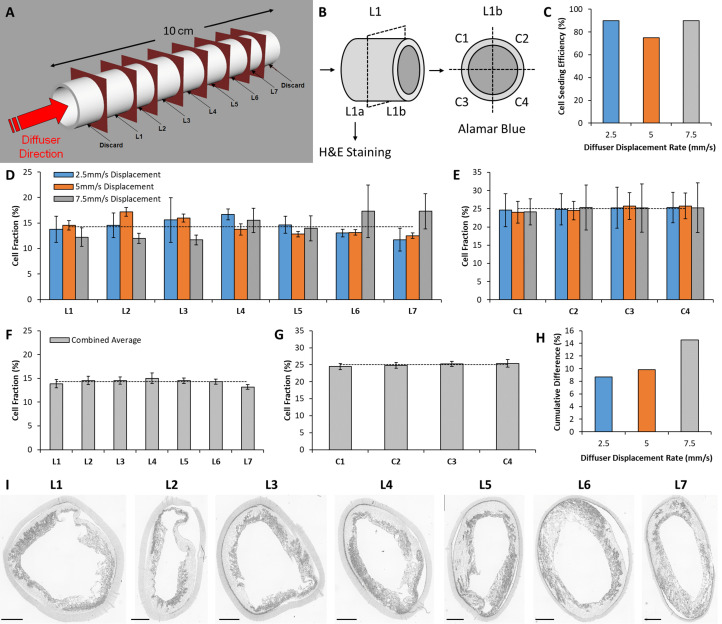
*In vitro* analysis of seeding device performance. **(A)** Schematic of the sectioning technique used to quantitatively assess the distribution of cells seeded within scaffolds using the novel seeding device. **(B)** Separation of each section for histological staining and metabolic activity assessment. Cell metabolic activity assay sections were further divided into quadrants to estimate circumferential cell distribution within the scaffold. **(C)** Scaffold seeding efficiency for each seeding configuration examined in this study. **(D)** Longitudinal distribution of cells across seven longitudinal sections for each seeding configuration. The dashed line indicates ideal cell distribution. **(E)** Circumferential distribution of cells across four quadrants for each seeding configuration. **(F)** Combined average of all three seeding configurations for longitudinal, and **(G)** circumferential distribution. **(H)** Cumulative difference between longitudinal cell distribution and the ideal distribution for each seeding configuration. **(I)** H&E staining of the seeded scaffold (seeded at a Diffuser displacement speed of 2.5 mm/s) to visualize the distribution of cells within the scaffold pores. Scale bars depict 1 mm.

After seeding, 1.5 cm of material was trimmed from the scaffold ends and discarded. Scaffolds were placed in 20 mL of GM and incubated overnight to facilitate cell adhesion under static conditions. Scaffolds were then sectioned into 1 cm segments and labeled L1 to L7 (Figure 2A). The segments were bisected longitudinally with one half reserved for histological staining (to visualize cell nuclei), and the other half reserved to assess cell metabolic activity (Figure 2B). Metabolic activity was used as a surrogate marker to assess the distribution of cells within the seeded scaffold.

#### Histologic Evaluation

Seeded scaffold segments intended for histological analysis were fixed in 4% paraformaldehyde and sectioned at a thickness of 10 μm using a microtome. Sections were stained with hematoxylin and eosin (H&E) to mark cell nuclei and determine hADMSC distribution in the scaffolds. Imaging was performed on a Nikon Eclipse 90i microscope (Nikon, Tokyo, Japan). Quantification of cell nuclei number was performed using the intensity threshold and particle count features available in ImageJ (Fiiji, public domain) (Soletti et al., 2011).

#### Metabolic Activity

Seeded scaffold segments intended for cell metabolic activity assessment were further sectioned into quadrants (labeled C1–C4) and each quadrant was placed in a well of a 48-well plate containing 500 μL of GM and 50 μL of Alamar Blue solution (Sigma). The quadrants were incubated for 4 h at 37°C, then removed from the Alamar Blue solution and squeezed to drain any remaining solution. The absorbance of the solution was read at 570 nm relative to 600 nm with a microplate reader (model 680, Bio-Rad, Hercules, CA, United States) and the absorbance of Alamar blue solution incubated with an unseeded scaffold section was subtracted as a blank control. The longitudinal distribution of cell metabolic activity was determined by pooling all four quadrants from the same longitudinal segment for segments L1–L7. The circumferential distribution of cell metabolic activity was estimated by pooling one quadrant from each longitudinal segment for quadrants C1–C4. However, circumferential distribution should only be regarded as an estimate as one quadrant from each longitudinal section was selected and pooled at random.

### *In vivo* Evaluation

#### Cell Source, Isolation, and Culture

Sheep stromal vascular fraction (sSVF) was selected as the cell source be to seeded within the PEUU scaffold intended for implant as we have previously demonstrated that the inclusion of such cells limits acute thrombosis and promotes positive vascular remodeling when incorporated into tubular PEUU scaffolds in a small animal model (Krawiec et al., 2017; Haskett et al., 2018). sSVF was obtained from the adipose tissue of a single donor sheep. Autologous cells were not used as sufficient adipose tissue could not be harvested from the same animal without seriously compromising animal health. Furthermore, our group has shown that autologous cells harvested from patients at risk of developing cardiovascular disease and requiring a bypass (e.g., diabetic or elderly patients) are not capable of generating viable TEVGs *in vivo*, with the implants predisposed to failure due to acute thrombosis (Krawiec et al., 2015, 2016).

The sSVF was isolated using previously described methods (Krawiec et al., 2017; Haskett et al., 2018). Briefly, adipose tissue was cut into approximately 10 cc portions and placed into separate 50 cc conical tubes. Each piece was minced and combined with a collagenase solution [Hanks’ Balanced Salt Solution (Invitrogen, Carlsbad, CA) containing 3.5% bovine serum albumin (Millipore, Charlottesville, VA) and 1 mg/mL collagenase type II (Worthington, Lakewood, NJ)]. Tubes were then incubated at 37°C with agitation for 1 h. Digested tissue was filtered through sterile gauze to remove undigested tissue fragments and then centrifuged at 400 × g for 10 min at ambient temperature. After centrifugation, the cell pellet was resuspended in an NH_4_Cl-based buffer (Beckman Coulter, Miami, FL) to lyse red blood cells. The resulting cell suspension was filtered through a 500 μm sieve and centrifuged at 400 × g for 10 min at ambient temperature. The resulting pellet was resuspended in defined culture media [1:1 Dulbecco’s modified Eagle’s medium (DMEM, Gibco) to DMEM/F12 (Gibco) with 10% fetal bovine serum (Atlanta Biologics), antibiotics (1% penicillin/streptomycin, 0.5% Fungizone, 0.1% gentamycin), and 10 μL/L dexamethasone] and plated in collagen-coated (rat tail, Sigma) 175 cm^2^ flasks. Upon becoming 80% confluent, the cells were removed from the plate using trypsin and expanded up to passage 4 in collagen coated 5-layer tower flasks using the same protocol outlined in section “Cell Source and Culture.”

#### Scaffold Implantation and Explant

Twenty four hours prior to implantation, a 12 cm PEUU scaffold was seeded with 200 million sSVF cells. The Diffuser speed was set to 2.5 mm/s for two cycles as this was shown to be the optimal displacement speed following validation testing (see section “Bulk Seeding Validation”). After overnight incubation in defined culture media, the scaffold was transported to the surgical facility and maintained in defined culture media until implantation.

The neck of a 9-month-old Suffolk sheep was prepared for surgery (Rojo Stock Farm, New Castle, PA). Prior to surgery, atropine (0.03–0.06 mg/kg), oxytetracycline (20–27 mg/kg), and heparin (5,000 IU) was administered. Morphine (0.2–0.5 mg/kg) was given as an analgesic and anesthesia was maintained with isoflurane (1.5–5% inhaled). A 14 cm incision was made in the skin running longitudinally along the medial border of the sternocleidomastoid muscle. A 10 cm portion of the carotid artery was isolated and exposed. The segment of isolated artery was clamped at each end using vascular clamps and an 8 cm length of carotid artery was excised. A 9 cm portion of the seeded scaffold was implanted as a carotid interposition graft. Both ends were oblique anastomoses sutured with a continuous running suture technique using 7-0 prolene sutures (Ethicon 8696G, Cincinnati, OH). The clamps were then removed, and the flow was confirmed using ultrasound. The muscle and skin layers were closed separately using a 2-0 and 3-0 vicryl running suture respectively (Ethicon J317H and J316H, Cincinnati, OH).

**FIGURE 3:**
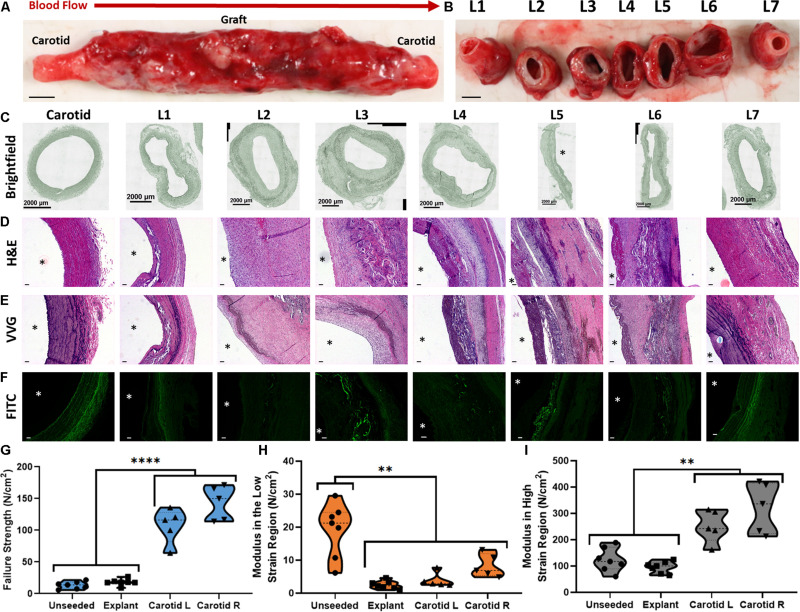
Large animal implantation and explant analysis of a large seeded construct. **(A,B)** Macroscopic image of the explanted TEVG (**A**, intact and **B**, longitudinally sectioned) after 10 weeks as a carotid interposition graft in a sheep model. Scale bars depict 5 mm. **(C)** Brightfield images of sections L1 to L7. **(D)** H&E staining of sections L1 to L7 demonstrating cellular distribution in the graft and neo-intima formation. **(E)** VVG staining of sections L1 to L7 demonstrating elastin distribution in the graft neo-tissue. **(F)** Auto-fluorescence of the PEUU material remaining in the graft. Scale bars depict 100 μm. **(G)** Failure strength, **(H)** modulus in the low strain region and **(I)** modulus in the high strain region for the unseeded scaffold, the explanted TEVG and the native carotid arterial tissue. ^∗∗^ indicates statistical significance at *p* < 0.01 and ^****^ indicates statistical significance at *p* < 0.0001.

After 10 weeks, the sheep was prepared for explant and euthanized as approved by the Institutional Animal Care and Use Committee. The initial incision site was re-opened, and the graft was isolated. The graft and a small portion of the proximal and distal carotid artery was harvested (Figure 3A). The graft was processed by sectioning it into seven separate segments (sections L1–L7) (Figure 3B). The segments were further bisected longitudinally with one half reserved for mechanical characterization and the other half reserved for histological staining.

#### Mechanical Characterization

Explanted graft ring segments intended for mechanical characterization were mounted on two parallel pins clamped within an Instron tensile tester (Instron, #5543A, Norwood, MA) and a preload of 0.01 N was applied. Each ring segment was extended at a displacement rate of 1 mm/min up to 20% strain for 5 cycles to precondition the tissue and then immediately extended until failure at the same rate. The mechanical properties of the explanted graft were compared to adjacent carotid artery tissue and unseeded PEUU scaffold material. The force-extension curves were converted to stress-strain curves using the following expressions (Macrae et al., 2016):

$$\text{Stress},\sigma =\frac{F}{2A_{\text{o}}};\text{Strain},\varepsilon =\frac{L+\pi r_{\text{w}}}{L_{\text{o}}+\pi r_{\text{w}}}-1$$

Stress and strain measurements are obtained from twice the original cross-sectional area (Ao) of the test specimen, the force (F) measured during each mechanical test, the sample gauge length in the loaded (L) and unloaded configuration (Lo), and the radius of the mounting pins (r_w_). Two separate moduli are calculated using the slopes of the linear portions of the mechanical response curve in the low- and high- strain regions. The transition point between low and high strain is defined as the point of the stress–strain curve with the maximum normal distance from the global secant, the line spanning from the origin to the end of the curve (Holzapfel, 2006; Cunnane et al., 2016). This equates to dividing the curve into three equal parts and treating the initial and final thirds of the curve as the low- and high-strain regions respectively.

#### Explant Staining

Explant segments intended for histological staining were sliced into 5 μm sections and stained for cell and elastin distribution using H&E and Verhoeff van Gieson (VVG) staining, respectively. Staining was performed by the Histology Core at the McGowan Institute for Regenerative Medicine. Stained sections were imaged using a Nikon 90i upright microscope. Prior to sectioning the explant for histological examination, the carotid artery tissue was separated from the TEVG along the anastomosis to ensure that sections L1–L7 reflect the structure of the explanted graft.

### Statistics

Statistical analysis was performed using GraphPad Prism 8 (GraphPad Software, San Diego, CA, United States). Data normality was examined using Shapiro-Wilk tests. Ordinary one-way ANOVA analysis was used to identify significant differences between more than two groups of variables. A *p*-value of less than 0.05 was considered statistically significant.

## Results

### Bulk Seeding Validation

Cell seeding efficiency for each diffuser displacement speed is displayed in Figure 2C. Efficiency was 90% for both 2.5 and 7.5 mm/s, and 75% for 5 mm/s. Figures 2D,E display the longitudinal and circumferential distribution of cell metabolic activity (section “Metabolic Activity”) within each scaffold, respectively. No intra-sample variance was observed for longitudinal or circumferential distribution (*p* > 0.05). The dashed lines in Figures 2D,E indicate the cell distribution within a theoretical sample that exhibits perfectly homogenous cell distribution (longitudinal = 14.29%, circumferential = 25%). The cell distribution of all samples is combined in Figures 2F,G to examine the pooled longitudinal and circumferential cell distribution over the three samples. The cumulative difference of each longitudinal section from the ideal distribution is displayed in Figure 2H for each diffuser displacement speed. The cumulative difference is 8.67, 9.81, and 14.57% for 2.5, 5, and 7.5 mm/s, respectively.

Figure 2I displays grayscale images of H&E staining that illustrate the cell distribution in sections obtained from the scaffold seeded at 2.5 mm/s. Qualitative assessment reveals the retention of cells within the scaffold structure. The cells are restricted to the inner layer of the scaffold and are unable to penetrate the outer layer due to the small pore size exhibited by the outer electrospun layer.

### Pilot *in vivo* Testing of a Cell Seeded TEVG Construct

The seeded construct was well-tolerated as a TEVG implant by the recipient animal over the 10-week period. The animal recovered fully from the surgery, demonstrating good health after 10 days and throughout the remainder of the study. Ultrasounds performed at weekly intervals confirmed longitudinal patency of the graft. Staining of the explanted graft revealed cell integration, scaffold degradation and neo tissue formation (Figures 3C–F).

H&E staining of all sections revealed cell-rich neo-intimal layer formation along the lumen of the explant (Figure 3D). VVG staining allows for differences in neo-tissue composition to be identified along the length of the scaffold. Ordered, stratified laminae of elastin can be seen in sections L1 and L7. Conversely, elastin is largely absent from sections L2, L3, and L6, while elastin appears in a disorganized fashion in sections L4 and L5 (Figure 3E).

Images depicting auto-fluorescence of the PEUU scaffold in the FITC channel reveal varying levels of scaffold degradation in each section (Figure 3F). It can be observed that sections L3, L4, and L5 exhibit the largest quantity of remaining scaffold material. This is intuitive as these sections represent the center of the scaffold which would be exposed to less endogenous enzymes capable of dissolving the PEUU.

Figures 3G–I display the mechanical characteristics of the unseeded scaffold, the explanted graft and the native carotid artery tissue. The data is presented as failure strength, and moduli in the low and high strain regions. The failure strength of the unseeded scaffold and the explanted graft are lower than the native tissue (*p* < 0.0001) (Figure 3G). However, the failure strength of the unseeded scaffold and explant are still above normal levels of physiological circumferential stress present in human common carotid arteries during diastole (13.37 ± 2.62 and 18.09 ± 2.21 vs. 6.3 ± 2.3 N/cm^2^) (Kamenskiy et al., 2012). Furthermore, no portion of the explant underwent rupture, highlighting the adequacy of the graft strength. The modulus of the explant in the low strain region is lower than the unseeded scaffold (2.5 ± 0.53 vs. 19.3 ± 3.77 N/cm^2^, *p* < 0.0001), indicating that the modulus decreases after the implant period (Figure 3H). This change in modulus is likely due to the resorption of the PEUU material coupled with the synthesis of elastic fibers, which combine to decrease the modulus of the graft to a more physiological level. The modulus of the explant in the high strain region remains similar to the unseeded scaffold due to the persistence of the electrospun layer (95.74 ± 9.51 vs. 123.64 ± 20.74 N/cm^2^, *p* > 0.05), but both are still below the values observed for the native tissue (253.1 ± 27.72 and 322.49 ± 43.17 N/cm^2^, *p* < 0.01) (Figure 3I).

## Discussion

We have successfully developed and validated a semi-automated device that allows for rapid, bulk seeding of large tubular scaffolds with cells. Furthermore, we have demonstrated the utility of this device by evaluating the *in vivo* remodeling of a cell seeded TEVG construct in a large animal pilot study. Validation of our device revealed successful seeding of hADMSCs throughout the full length of our PEUU scaffolds with homogenous longitudinal and circumferential cell distribution. Using our validated cell seeding device, approximately 200 million sSVF cells were incorporated into a 12 cm PEUU scaffold, which was cut to 9 cm and implanted as a carotid interposition graft in a sheep model. The seeded graft remained patent, exhibited signs of scaffold degradation and initiated neo-tissue formation throughout the length of the graft after 10 weeks *in vivo*.

Static, quasi-static, rotational, bioreactor and vacuum cell seeding approaches have all been previously employed to incorporate cells into tubular scaffolds for the purpose of developing TEVGs (as reviewed in Weinbaum et al., 2020). Our rotational, vacuum cell seeding approach offers considerable advantages over alternative cell seeding techniques. Rotational, vacuum seeding achieves much higher seeding efficiencies and more even cell distributions compared static loading (Hibino et al., 2010; Roh et al., 2010; Lee et al., 2016), does not require the cell or scaffold modifications that quasi-static seeding techniques necessitate (Tiwari et al., 2003; Sagnella et al., 2005; Perea et al., 2006; Shimizu et al., 2007; Campagnolo et al., 2016), is far faster than bioreactor techniques (Sodian et al., 2002; Zhao and Ma, 2005; Inoguchi et al., 2007; Melchiorri et al., 2016), and offers improved cell distribution and cell viability compared to techniques that employ exclusively rotational (Nasseri et al., 2003; Godbey et al., 2004; Hsu et al., 2005; Aper et al., 2016) or vacuum approaches (van Wachem et al., 1990; Udelsman et al., 2011; Kurobe et al., 2015). This study advances the current state-of-the-art for rotational, vacuum cell seeding by scaling our cell seeding technology into a novel semi-automated seeding device that is capable of fabricating reproducible human-sized, cell-seeded TEVG constructs. The technology presented herein therefore has considerable implications for TEVG research that incorporates cells into tubular scaffolds, as our semi-automated seeding approach and promising pilot *in vivo* data increase the likelihood of securing regulatory approval and effective clinical translation of our TEVG technology.

This present study has a number of limitations. Firstly, the pilot study only performs a single implantation. The primary objective of this manuscript was to describe our novel seeding device and validate it by determining if it can produce cell seeded TEVG constructs of a size necessary for human (and large animal) implantation. We then performed an initial pilot implantation study as a secondary objective to investigate if our novel seeding device can produce TEVGs that do not undergo acute thrombosis and initiate neo-tissue formation *in vivo*, prior to transitioning toward a multi-animal study. Future studies will increase implant numbers to examine the reliability of this approach and build confidence toward a dependable clinical treatment option. Secondly, longer explant time points are required to provide a more robust picture of graft longevity in terms of sustained patency and long-term remodeling. The presence of organized elastin at the edges of our explanted TEVG, and the absence of organized elastin toward the center of the graft, suggests that the seeded construct is still undergoing remodeling, and that longer time point studies are required to fully characterize neo-tissue formation and mechanics once the scaffold has completely remodeled. However, the presence of physiologically relevant elastin formation in the outer sections of the explant is promising for the future of our cell seeding device and TEVG technology. Furthermore, it has previously been shown that the PEUU material used to fabricate the scaffolds in this study undergoes an approximate 50% reduction in molecular weight over an 8-week period (Guan et al., 2002), and the presence of PEUU mass at 10 weeks (Figure 3) further suggests that longer time point studies are required to characterize the remodeling of our TEVG up to the point where all of the scaffold material has been resorbed. Future studies should therefore seek to characterize explanted TEVGs after at least 6 months *in vivo* to better understand the effects of vascular remodeling and scaffold degradation on long-term graft viability. Finally, our construct was implanted as a carotid graft which is a straight configuration. However, many bypass grafts in clinical practice must be bent and curved around obstructions. Future studies must demonstrate the grafts ability to withstand kinking in complex configurations *in vivo* (Van Epps et al., 2009) or the possibility to modify the scaffold structure in order to mitigate mechanical instability such as kinking or buckling.

With respect to future practice, this study brings TEVG technology closer to clinical translation in two ways. Firstly, the development of our seeding device will allow us to create and test clinically relevant grafts in a reproducible manner, thus accelerating the clinical translation of small-diameter arterial TEVGs. Secondly, the human-sized TEVG that we generated in this pilot study using our scaffold-seeding technology undergoes full cellularization, partial degradation and positive matrix remodeling after 10 weeks *in vivo* therefore demonstrating that our novel TEVG cell-seeding technology warrants further investigation via increased implant numbers and longer explant time points to fully understand the clinical potential of this promising treatment modality.

## Conclusion

Our novel, semi-automated, bulk seeding device makes it possible to rapidly generate “human-sized” cell-seeded, tubular TEVG constructs. Our *in vitro* results demonstrate that the novel device allows for uniform longitudinal and circumferential cell seeding. Our pilot *in vivo* results demonstrate graft patency, scaffold degradation and neo-tissue formation within the TEVG seeded construct using the presented device. The findings of this study support our hypothesis that it is possible to use an automated system to generate “human-sized” TEVGs capable of positive vascular remodeling in a large animal model.

## Data Availability Statement

The raw data supporting the conclusions of this article will be made available by the authors, without undue reservation, to any qualified researcher.

## Ethics Statement

The animal study was reviewed and approved by the University of Pittsburgh Institutional Animal Care and Use Committee.

## Author Contributions

EC and KL: concept generation, literature review, experimental work, and manuscript writing. LS, ET, ADŠA, and SL: concept generation, experimental work, and critical review of the manuscript. AR: experimental work and critical review of the manuscript. DH: concept generation, literature review, experimental work, and critical review of the manuscript. WW, JW, and DV: concept generation and critical review of manuscript. All authors contributed to the article and approved the submitted version.

## Conflict of Interest

The authors declare that the research was conducted in the absence of any commercial or financial relationships that could be construed as a potential conflict of interest.

## Abbreviations

DMSO: dimethyl sulfoxide
FBS: fetal bovine serum
H&E: hematoxylin and eosin
hADMSC: human adipose derived stem cell
PBS: phosphate buffered saline
sSVF: sheep stromal vascular fraction
TEVG: Tissue engineered vascular graft
VVG: Verhoeff van Gieson.
