# Supplementary material for: Development of a Semi-Automated, Bulk Seeding Device for Large Animal Model Implantation of Tissue Engineered Vascular Grafts
Source: Front Bioeng Biotechnol. 2020 Oct 23;8:597847. doi: 10.3389/fbioe.2020.597847 (PMC7644804; doi:10.3389/fbioe.2020.597847)
Supplement: Supplementary file 1 [file Data_Sheet_1.pdf]

Supporting Information

Development of a Semi-Automated, Bulk Seeding Device for Large Animal Model Implantation of Tissue Engineered Vascular Grafts

Eoghan M Cunnane*[1,2], Katherine L Lorentz*[1], Lorenzo Soletti[1], Aneesh K Ramaswamy[1], Timothy K Chung[1], Darren G Haskett[1], Samuel K Luketich[3], Edith T Tzeng[4], Antonio D'Amore[3], William R Wagner[3,5], Justin S Weinbaum[1,3,6], David A Vorp[1,3,4,5,7,8]

[1] Department of Bioengineering, University of Pittsburgh, Pittsburgh, PA, US
[2] Tissue Engineering Research Group, Dept. of Anatomy, Royal College of Surgeons in Ireland, Dublin, Ireland
[3] McGowan Institute for Regenerative Medicine, University of Pittsburgh, Pittsburgh, PA, US
[4] Department of Surgery, University of Pittsburgh, Pittsburgh, PA, United States
[5] Department of Chemical and Petroleum Engineering, University of Pittsburgh, PA, US
[6] Department of Pathology, University of Pittsburgh, Pittsburgh, PA, United States
[7] Department of Cardiothoracic Surgery, University of Pittsburgh, Pittsburgh, PA, US
[8] Clinical & Translational Sciences Institute, University of Pittsburgh, Pittsburgh, PA, US

* Denotes joint first authorship

Address correspondence to:
David A. Vorp, Ph.D.
e-mail: vorp@pitt.edu

1 Supplementary Text

1.1 Design of the translating-rotating seeding device

Our novel device achieves bulk seeding of human-sized scaffolds via local delivery of cells through a mobile internal sliding body capable of releasing cells locally in a controllable manner. The system includes a cell releasing “Diffuser” equipped with radial nozzles attached to an arm or “Stylet”. The Stylet drives the Diffuser along the longitudinal axis of the tubular scaffold and also transports the cell suspension from a syringe to the scaffold’s lumen while the scaffold rotates under a vacuum, **Figure 1A**. The basic principle of the device includes rotation, vacuum, and infusion of the seeding suspension via a syringe pump. The locally delivered cells are drawn into the wall of the scaffold, via the applied vacuum, through the interconnected pore network, to ensure even radial cell distribution. The linear displacement of the Diffuser ensures even longitudinal cell distribution, while the rotation of the scaffold ensures even circumferential distribution.

Linear translation of the Stylet/Diffuser is achieved using a stepper motor attached to a sliding stage. A central screw attached to the stage converts the rotational movement of a stepper motor (“Motor 1”) (S57-83, Parker Compumotor, Rohnert Park, CA) to translational movement. A 3D printed Stylet holder is mounted on the sliding stage to transmit translational motion from the sliding stage to the Stylet. The Stylet was fabricated from a SS 316L tube (OD = 3.175 mm, ID = 0.98 mm, length = 25.4 cm). The Diffuser was machined from a single PTFE cylindrical rod (OD = 3.175 mm, length = 5 mm). A central lumen was created through 3/4 of the Diffuser’s length and eight equi-spaced radially-distributed bores (Ø = 0.62 mm) were drilled to allow for even diffusion of the cell suspension. Friction between the Diffuser and the scaffold is prevented during infusion by under sizing the Diffuser so that it does not come into contact with the inner lumen of the scaffold (ID = 4.7 mm).

Rotation of the scaffold is achieved using an additional stepper motor (“Motor 2”) connected to a mounting tee located distally to the scaffold (“Driving Tee”). The Driving Tee is connected to the proximal mounting tee (“Driven Tee”) via a bracket that transfers the rotational motion. Both tees consist of a 7 cm-long SS 316L tube (OD = 6.35 mm, ID = 5 mm) and allow for concentric sliding of the Stylet within the internal lumen of the Driven Tee, **Figure 1A**. Two PTFE cylindrical tips (Ø = 5 mm; length = 1.5 cm) are attached to the ends of the mounting tees to allow for concentric alignment of the Stylet within the Driven Tee and mounting of the scaffold within the vacuum chamber, **Figure 1B-C**. Two SS 316-L inline swivel joints (007-10211-ZAT, Rotary Systems, Inc., Ramsey, MN) are used as rotating joints to allow for sliding of the Stylet while the Driven Tee rotates. A fluoroelastomer V-ring seal (SKF, Göteborg, Sweden) is used to create an airtight seal between the translating Stylet and the interior of the chamber. The rotating joints are mounted to the base using two adjustable supports (Delrin®) to accommodate scaffolds of varying length. The Driving Tee has a polypropylene Luer Lock female port attached to the rotating joint to close the circuit downstream after priming of the scaffold, **Figure 1D-E**.

A power supply (XL-PSU, Parker Compumotor, Rohnert Park, CA) is used to energize two digital micro-stepping drivers (ViX IM250, Parker Compumotor, Rohnert Park, CA) that provide motion signal to the motors. The drivers are connected to each other via an RJ-45 cable and to a PC via an ethernet cable. The syringe pump is connected to the PC via a Labview card. Both the syringe pump and the motors

are controlled via a custom Labview program that allows for control of the Stylet translation speed, the scaffold rotation speed and the syringe pump infusion.

All of the components of the device that come in contact with the scaffold or the cells during the cell seeding procedure (chamber, support rods, torque transfer bracket, infuser rod and syringe-connection-tube) were sterilized via ethylene oxide prior to seeding each scaffold with cells.

1.2 PEUU Scaffold imagine

Figure S1 displays luminal and abluminal images of the scaffold inner layer, prior to the application of the electrospun outer layer, obtained using a scanning electron microscope. The pores on the luminal surface allow for infiltration of cells throughout the wall of the scaffold inner layer during seeding.

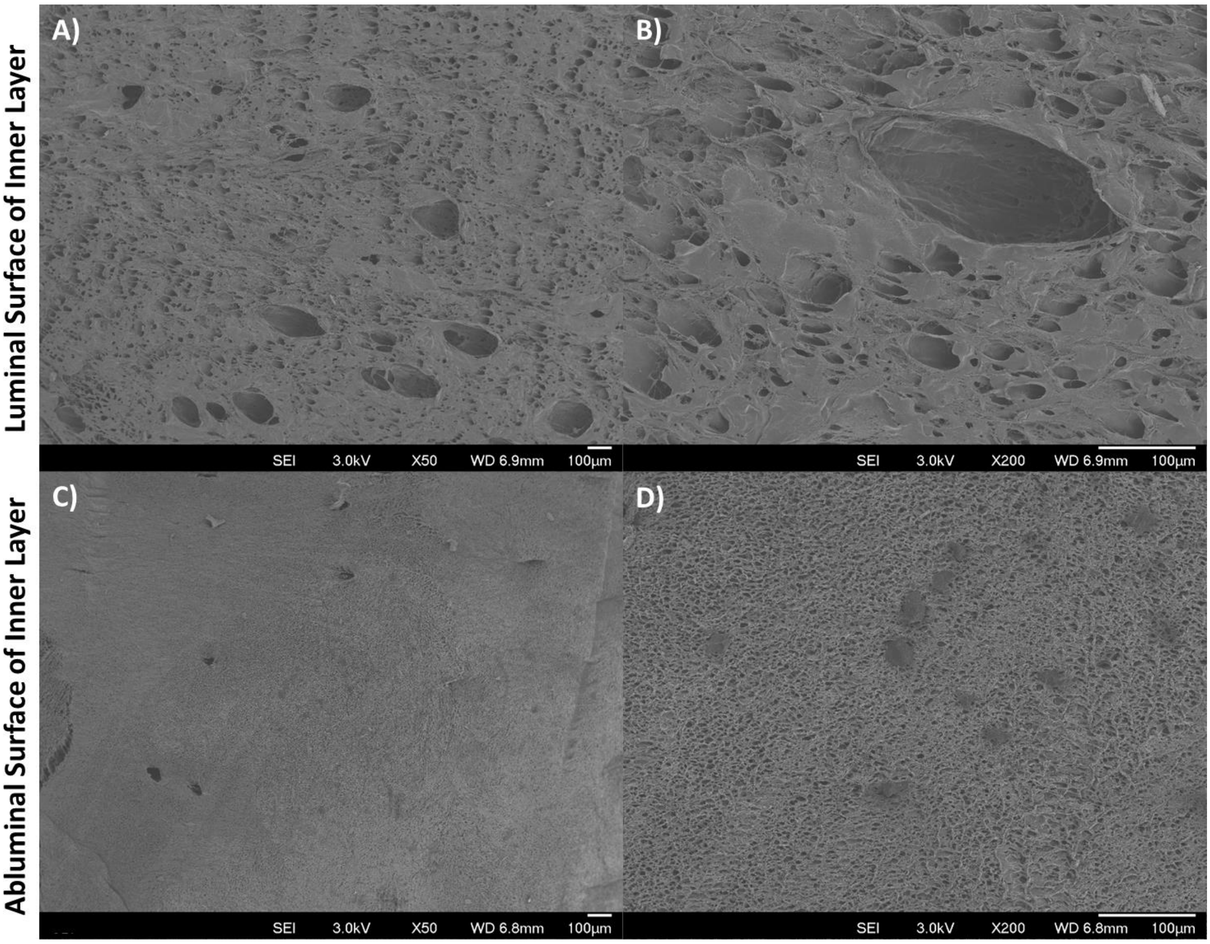

Figure S1: Scanning electron microscope images of the scaffold inner layer. A and B) Luminal images of the scaffold inner layer at 50x and 200x magnification. C and D) Abluminal images of the scaffold inner layer at 50x and 200x magnification.

2 Supplementary Calculations

2.1 Shear stress estimation

The seeded cells are administered in cell culture media (with dynamic viscosity, μ of 1.03×10^{-3} Pa.s) through the stylet (with an inner radius, R of 4.9×10^{-4} m) at a flow rate, Q of 12.5 ml/min (or 2.083×10^{-7} m^3/s). The shear stress on the cells, τ_S, can be calculated using Poiseulle's law,

$$\tau_S = \frac{4\mu Q}{\pi R^3}$$

as 23.2 dynes/cm^2, a value that is within the range of wall shear stress that occurs in arteries (10 to 70 dynes/cm^2) (Malek et al., 1999). The level of shear stress applied to the cells during seeding was therefore deemed to be acceptable.

2.2 Cell seeding density

The dimensions of rat aorta sized scaffolds were obtained from Soletti et al., 2011 and the cell density of the rat and sheep sized scaffolds are detailed in **Table S1**.

Table S1: *Parameters and values used to calculate the cell density within the rat aorta and sheep carotid sized scaffolds.*

Scaffold Size	Length (cm)	External Radius (cm)	Internal Radius (cm)	Vol (cm^3)	Cell Number	Cell Density (cells/cm^3)
**Rat Aorta**	1	0.08	0.065	0.006833	3M	439
**Sheep Carotid**	12	0.26	0.235	0.466527	200M	428

2.3 Cell number estimation

This section details the calculations performed to estimate the cell number present in each scaffold segment and histological section following cell seeding. The seeding efficiency achieved for each seeding run was either 75% or 90%. Therefore, if we take the case of 90% seeding efficiency, the total number of cells actually seeded into the scaffold can be estimated as 0.9 x 200 million = 180 million, as the total number of cells in the suspension was 200 million cells. If we consider that 9 cm of the scaffold's length is available for seeding (considering that 0.5 mm at either end of the scaffold is mounted), then approximately 20 million cells will be seeded per cm of scaffold, or 140 million cells will be contained in the 7 cm length of scaffold that we examine. As only half of every 1 cm segment is subjected to Alamar blue analysis, then it can be estimated that the cumulative number of cells contained within the seeded scaffold segments depicted in Figure 2D-F is 70 million cells. Similarly, if we consider that each histological section is 10 μm, then it can be estimated that there are approximately 20,000 cells visible in each section of Figure 2I. Figure S2 below depicts an alternate Figure 2 where the parts D-F depict the estimated cell number in each scaffold segment, rather than the cell fraction in each segment.

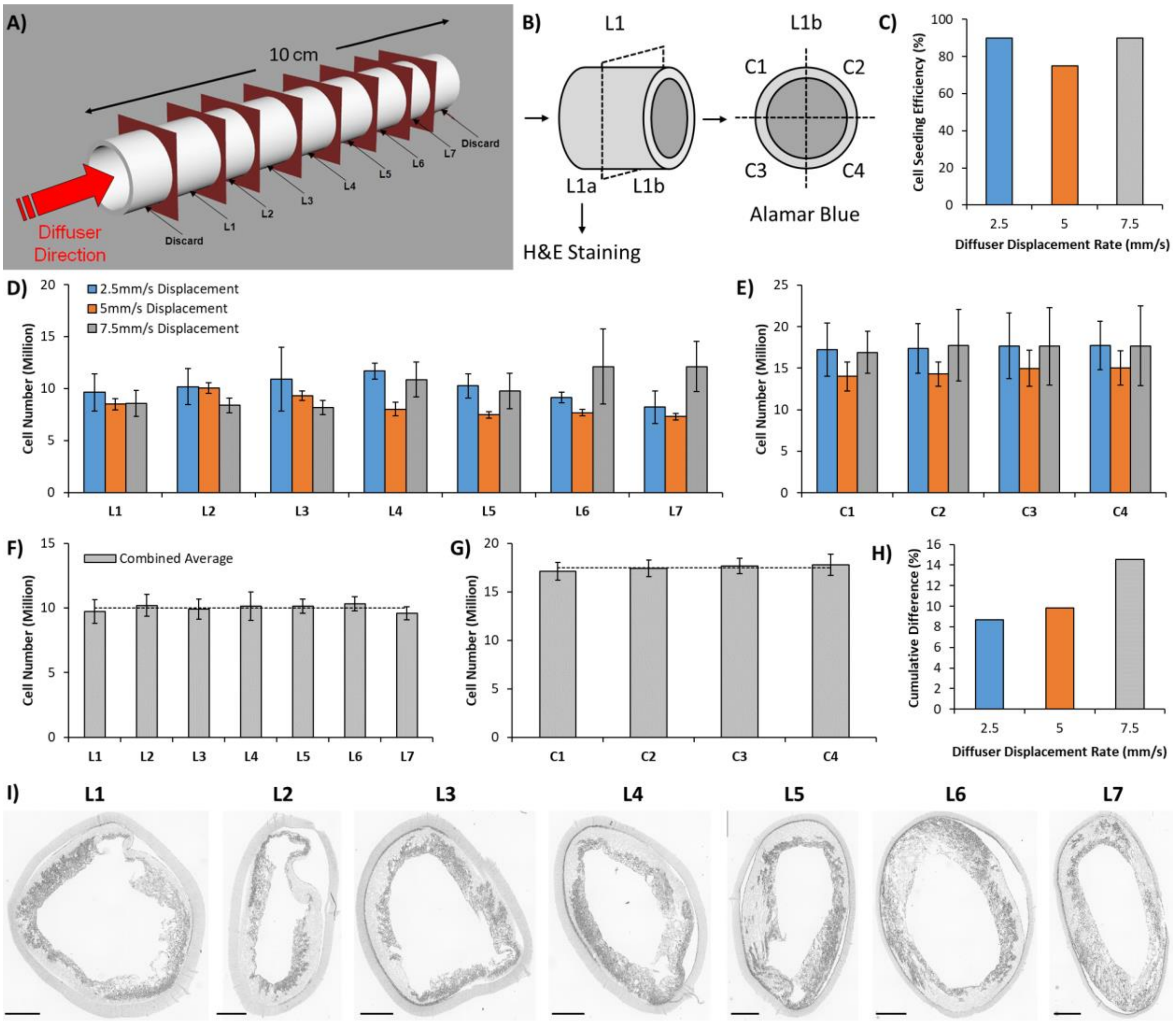

Figure S2: In vitro analysis of seeding device performance. A) *Schematic of the sectioning technique used to quantitatively assess the distribution of cells seeded within scaffolds using the novel seeding device.* ***B)*** *Separation of each section for histological staining and metabolic activity assessment. Cell metabolic activity assay sections were further divided into quadrants to estimate circumferential cell distribution within the scaffold.* ***C)*** *Scaffold seeding efficiency for each seeding configuration examined in this study.* ***D)*** *Longitudinal distribution of estimated cell number across 7 longitudinal sections for each seeding configuration.* ***E)*** *Circumferential distribution of estimated cell number across 4 quadrants for each seeding configuration.* ***F)*** *Combined average of 3 seeding runs (all with 90% seeding efficiency) for longitudinal and* ***G)*** *circumferential distribution.* ***H)*** *Cumulative difference between longitudinal cell distribution and the ideal distribution for each seeding configuration.* ***I)*** *H&E staining of the seeded scaffold (seeded at a Diffuser displacement speed of 2.5 mm/s) to visualize the distribution of cells within the scaffold pores. Scale bars depict 1 mm.*

3 References

Malek, A. M, Alper S. L., Izumo, S. 1999. "Hemodynamic Shear Stress and Its Role in Atherosclerosis." JAMA, 282(21), 2035. doi:10.1001/jama.282.21.2035

Soletti, L., Nieponice, A., Hong, Y., Ye, S. H., Stankus, J. J., Wagner, W. R., and Vorp, D. A. 2011. "In Vivo Performance of a Phospholipid-Coated Bioerodable Elastomeric Graft for Small-Diameter Vascular Applications." Journal of Biomedical Materials Research - Part A 96 A (2): 436–48.
